# Etiology of Drug Abuse: A Narrative Analysis

**DOI:** 10.1155/2014/352835

**Published:** 2014-08-26

**Authors:** Nadjme Jadidi, Nouzar Nakhaee

**Affiliations:** ^1^St. Vincent Hospital, St. Vincent Health, Melbourne, Australia; ^2^Neuroscience Research Center, Institute of Neuropharmacology, Kerman University of Medical Sciences, Kerman, Iran

## Abstract

*Introduction and Aim*. Further gains in the prevention of drug abuse disorders require in-depth and holistic understanding of the risk factors of addiction from different perspectives. Lay persons and experts have different concepts of risk which could complement each other. The purpose of this study was to elaborate drug abuse risk factors through the story of individuals who had become drug dependent. *Design and Methods*. In this qualitative research, 33 individuals attending treatment centres for drug abuse were interviewed about the story of their addiction in Kerman, Iran. Interview questions were around the story of the participants. *Results*. All participants were male and in the age range of 18–40 years. Narrative analysis identified five themes as the main risk factors: family factors, peer pressure, the effect of gateway drugs (especially waterpipe), individual characteristics, and the community factors. More emphasis was placed upon the role of family factors, peer influence, and gateway effect. *Discussion and Conclusion*. This study elicited information from drug dependent subjects regarding the risk factors of drug abuse. According to drug dependent individuals' views, more attention should be devoted to family and peer influences by policy makers, in developing culture-based preventive strategies.

## 1. Introduction

According to the UNODC report, approximately 5% of the world adult population have used illegal drugs at least once in 2010 and 0.6% of people are considered “problem drug users” [1]. Drug abuse will impact various aspects of one's life including physical, mental, and social aspects. In addition to more than 200,000 deaths per annum due to heroin and cocaine abuse, drug abuse could lead to delinquency, early sexual activity, family disintegration, and increased risk of HIV [1, 2]. Although the rate of drug abuse is reported to be steady in some countries, it has shown an increasing trend in many developing societies [1]. Iran is facing an increasing number of drug abusers that have negative social and health impacts [3].

Over the past century, many theories have been proposed to describe the aetiology of drug abuse [2, 3]. These theories are categorised in 3 main subgroups of social, psychological, and biological subgroups [4]. Although multiple theories would help in better understanding of the aetiology of addiction, this multiplicity could convey the lack of consensus around aetiology of drug abuse [2] and, according to Spooner, scientific evidence in this area is inadequate [5].

The majority of studies in drug dependency have a quantitative approach that compares some of the factors in drug abusers with those of the nonusers [6, 7]. Considering lack of adequate knowledge about predisposing factors for addiction, experts recommended the use of “new models of risk factor research” [7]. Due to the complex nature of drug dependency, qualitative studies could be beneficial in exploring the process of addiction [3, 8]. Through deeper understanding of drug abusers, qualitative studies could throw light on why some people abuse drugs [8]. Narrative enquiry is a relatively new method in qualitative studies mainly used in the field of social science [9]. In this method, data collection is done through story telling [10]. Story is a rich source to obtain better insight into the social process [11]. Through learning how people become addicted, one could probably better understand “why they engage in these unhealthy behaviours” [12]. Narrative analysis has been used to discover the circumstances surrounding events such as injuries and maternal death [13]. To the best of our knowledge this method has not been used in the field of addiction etiology. Nevertheless, hearing people's story of their addiction could give us some cultural information and also assist us in prioritizing preventative activities as a supplement to research and hence it could increase the practicality of our findings. Solvic, in his famous article “Perception of risk” with more than 4500 citations, believes that the layperson's opinion should be considered in the design of preventative interventions as their opinion is frequently regarded as a supplement to the experts' view [14]. He even states that “their basic conceptualisation of risk is much richer than that of the experts” [14]. The purpose of this study was to expand on the existing body of literature by exploring the interview narratives of drug abusers.

## 2. Methods

The study was approved by the ethics committee of Kerman University of Medical Sciences. Subjects were individuals referred for detoxification or maintenance therapy to addiction treatment centres in Kerman city, the capital city of the largest province in Iran. The reason these subjects were chosen was that the focus of our study was on explanation of drug abuse risk factors rather than drug use. It was expected that individuals who experienced drug abuse would be able to provide richer accounts of “drug abuse” risk factors comparing to those with infrequent “drug use” [15]. This distinction should be considered by the researchers working on the aetiology of addiction [6]. All participants had already passed the detoxification phase and were in a stable condition. The sampling technique was purposive sampling.

Interviews were conducted after obtaining informed consent and ensuring confidentiality and anonymity. Interviews were performed in a private and quiet room. Each interview took between 30 and 90 minutes. All interviews were tape-recorded and then transcribed. Interviews started with a general question and then carried on according to the participants' responses.

Interview questions were around the story of the participants' drug abuse from the experimental stage through to abuse. The interview was started with a question about their own background and then a follow-up question, “Tell me about your first experience with drugs?” Three dimensions of the metaphoric narrative inquiry were considered by the interviewer (Nouzar Nakhaee). Those three dimensions of drug abuse were place, time sequence, and social and personal interaction [9].

Narrative analysis was constructed through pragmatic method as the purpose of narrative inquiry was not to create story for the outcome of research but to extract and categorise risk factors for drug abuse based on the content of the story [16]. Risk factor is defined as “an individual attribute, individual characteristic, situational condition, or environmental context that increases the probability of drug use or abuse or a transition in level of involvement in drugs” [17].

Transcripts were broken into small units through analysis of the content of the stories [18, 19]. We used Labov's method of transcription to construct a text from the interviews [20]. This method is one of the approaches for organizing narrative data which is useful for understanding major events in the life histories [20]. Primary codes (risk factors) were categorised based on similarity and formed into subcategories. Finally, the main themes were identified through making different subcategories.

## 3. Results

Thirty-three interviews were conducted. All participants were male and in the age range of 18–40 years. Only 2 participants had university qualifications. Findings were categorised in 5 themes as follows.

### 3.1. Family Relationships and Structure

Parental discipline style was one of factors that participants focused on.

*“My father used to beat me and I was stubborn and would come home even later at night. Then he would beat me harder and I became more stubborn.”*

*“I didn't dare talking to my father to tell him about my wishes. Our parents' thoughts were focused on earning money to buy a house, a car, etc. rather than making time for their children and to respecting their identity.”*

Lack of a warm and emotionally rich environment at home was another factor extracted from the participants' stories.

*“I can't recall if my parents have ever hugged me. I felt an emotional emptiness.”*

*“An addict wouldn't in the first place go towards drug abuse out of leisure. You don't become an addict if you were supported by the family.”*

*“I would rather play cards with my dad than my friends.” *

Another factor was noted as lack of supervision by parents.

*“This wouldn't have probably happened if my parents hadn't left me on my own.”*

*“If one day I had children, I would shadow them all the time and not leave them on their own.”*

Another risk factor was identified as copying parents especially the father.

*“I remember my dad smoked opium and I wished I could do the same when I grew up.”*

*“My father was an opium smoker. I thought he is an adult so he is doing the right things so if I smoked opium like him, I would grow up and become an adult like him.”*

Disrupted/disintegrated family structure was another risk factor.

*“Peace disappeared from our home when mum died and dad re-married so I smoked opium to keep calm.”*

*“When dad went to prison, there was no one to look after me, to keep an eye on me and to buy me clothes, stationary, etc.”*

Reviewing the content of stories, family relations were the most prominent and frequent theme amongst all the themes explored from the interviews.

### 3.2. Peer Influence

Peer pressure was emphasised by the interviewees as a main factor in experimenting drug abuse, and especially turning to heavier drugs. Some identified that peer pressure and engaging with the wrong crowd would stem from their own personality that would innately attract this type of people.

*“My addiction started with partying and night life.”*

Some identified their linkage with the wrong crowd as an accident.

*“We had a new neighbour whose son was my age. He was the first person introducing me to cigarettes and porn movies.”*

Some mentioned being teased and criticised by friends as the main factor for drug abuse.

*“I didn't want to smoke opium but my friends would tease me and say you are a sook. Why don't you smoke you little nerd?”*

Some identified the main factor as positive expectation from friends.

*“One of my friends suggested I smoke heroin to beat my competitor in Kong Fu matches.”*

### 3.3. Individual Characteristics

In this study, individual characteristics were identified as one of the risk factors. Starting drug abuse at a young age was observed in many participants in the way most of them reported their first time use during primary school. Particularly participants who started with cigarettes or waterpipe identified pleasure being the main reason for drug abuse.

*“I met a girl and I stayed smoking opium to help delay ejaculation.”*

Some participants identified their personality problems as the main risk factor in turning towards drugs.

*“I was aloof and timid and I always saw myself less than others.”*

*“I always looked for someone who would help me with my loneliness.”*

*“I would feel like a man when I smoked cigarettes.”*

*“I wouldn't take any advice and always wanted to experience things myself.”*

*“I thought addiction was for others and I would be able to control myself.”*

Some individuals mentioned denial as a mechanism and a reason to continue on their drug abuse.

*“We, addicts, are the last ones who realise that we have become dependent.”*

Some identified hereditary factors.

*“I feel this has been my fate because I had addiction in my genes just like diabetes that shows a few years later down the track.”*

*“If parents are addicted, the zygote is contaminated and the child would become dependent even with minimal use over a short period of time.”*

### 3.4. Gateway Effect

One concept extracted from the interviews was that problem drug abuse is a transition from lighter use such as waterpipe, cigarettes, hashish, and alcohol. Some individuals identified biological factors as the main risk factor in this transition.

*“My very first time use was with waterpipe. Waterpipe releases a code that is, you are no more scared of smoking. Although it is only nicotine, it would open the gate to other drugs.”*

Some participants identified the gateway effect through social factors.

*“I started with waterpipe but then changed to cigarettes as it was easier to hide.”*

*“Smoking waterpipe would predispose you to smoke opium just for the sake of being around together with others smokers.”*

Overall, out of all 4 drugs, there was more emphasis on cigarette and waterpipe compared to alcohol and marijuana. The majority of participants did not report their first experience as being with opium and/or heroin.

### 3.5. Community Influence

Some individuals identified more environmental risk factors such as poverty, type of neighbourhood, lack of leisure facilities, and normalised attitude towards drug abuse.

*“We didn't have facilities for healthy activities so we would gather together in a vacant house and drink alcohol.”*

*“Drugs were easily available around our neighbourhood just like lollies. How could you not get affected?”*

*“I got involved with drugs when my father was prisoned and we became poor and had to deal with poverty.”*

*“Many restrictions in the society such as the police being strict on the single male population in the parks and on the streets would make us go and gather in quiet places.”*

*“I always envied having a nice school bag or a proper outfit.”*

*“People told me that hey, everyone smokes, why you don't? How long do you want to live?”*

## 4. Discussion

This research elicited information from lived experience of drug dependent individuals of Iran regarding the risk factors of drug abuse. In this study, we categorised drug dependency risk factors in 5 main themes of which 3 were common in most of interviews, that is, role of the family, peer pressure, and starting drug abuse with lighter items such as cigarettes, waterpipe, and alcohol. The major limitation of this study was the fact that generalizability to other settings especially Western culture may be problematic and its main advantage was the ability to illuminate the inner life and live experiences of drug abusers.

Prevention is the most cost-effective method to address drug abuse [20] and recognition of drug abuse risk factors is required to design preventative interventions [3, 7]. However, some countries have built up their main services focusing on harm reduction or war on drugs [21]. In order to extract and interpret the risk factors, it is important to know that risk factors are not in isolation from each other and they do not work in a vacuum but are rather related to each other so we should be careful not to have a narrow, parochial, and dogmatic approach in identifying and interpreting those risk factors [6].

On the other hand, we need to be aware that designing preventative interventions for drug abuse would probably be unsuccessful without considering the drug dependent population's views [22]. Qualitative studies are good at finding the blind spots of some phenomena through in-depth interview. These blind spots are usually not detectible through quantitative studies. In this study, narrative analysis was used to explore the risk factors of engaging in unhealthy behaviours from the drug abusers' point of view. Furthermore, these risk factors exist within the culture of a society in which drug abusers live. Given the numerous theories and risk factor overload, this study was helpful to refine the more important risk factors in a culture-based framework.

In this study, all participant identified family as the most important and crucial risk factor for drug abuse in the youth. Amongst all the family risk factors, some were more prominent such as physical punishment, lack of a safe and warm environment at home, weak attachment between children and the parents, role modelling of the parents, and loss of a parent. Lack of supervision and monitoring of children by the parents were also identified amongst the risk factors extracted from the interviews. Overall, family relationships had a more significant role than that of the family structure. Family can play a role in development of drug abuse in different ways. Lack of a warm and supportive environment would increase the risk of disruptive and unhealthy behaviour in children [6]. History of adverse childhood event especially before the age of 5 can substantially increase the risk of drug dependence in adolescents [23, 24]. Also, neglectful parents do not supervise their children's choice of friends [6]. Weak parent-child attachment would lead to strong bonding with friends resulting in peer pressure in youth [4]. Owing to modernization, integration of women in the work world, and reduction of family size, Iranian family is passing through a fast transition that is becoming more similar to Western family. Family's educative function has been delegated to television, internet, school, and other institutions so the supportive role of families seems to be fading. All these factors may provoke the risk of drug abuse by adolescents due to a lack of strong bond between parents and child.

According to theories of social learning and social control, the stronger the family cohesion and parental monitoring are, the less probable the drug abuse and turning to gateway drugs are. Parents role modelling, also identified by the participants, has been proved in various studies [7, 25]. Culture-based family education programmes to reveal the pivotal role of families in prevention of youth drug abuse would have potential benefits for community [23, 24].

In this study, peer pressure has been identified so strongly that most of participants mentioned the stereotype statement “I wouldn't become addicted if I didn't have bad friends.” Almost all studies agree on the impact of peer-related factors [7], but some would clearly recognise it as the strongest risk factor for drug abuse in youth [26, 27].

Regarding how an individual adolescent would go on a socialization pathway through a friend, some experts believe that potential drug abusers do not accidentally fall into peer clusters, but they are attracted through their common rules and similar attitudes known as “selective recruitment” [4]. However, some regard this as a casual process and identify the main factor being lack of supervision by parents in the process of friend finding which leads to adolescents being deceived by their peers [20, 28]. This study was more in line with the latter. Our study revealed that peer variables are among the strongest predictors of adolescents' drug abuse and parental neglect and lack of supervision may have a crucial role in triggering the peer influences [3, 27, 28]. As a whole, the role of peer factors needs to be emphasized in teaching programmes for both youth and parents.

In general, family and friend factors always interact, but it could be speculated that, in developing countries with less modernisation effect, children are more strongly bonded with the family so they would be more impacted by the family in both positive and negative ways [3].

Most of participants reported the experience of transition from drug abuse to problem drug abuse, that is, from lighter drugs such as cigarette, waterpipe, and alcohol to heavier drugs. Unlike the general consensus on the role of family and peer pressure, there was not an agreement on the gateway effect [4, 6] and some even recognised it as a cultural myth [7].

In our study, waterpipe has been mentioned as an important gateway drug which could be due to high rates of waterpipe use in Iranian young population [29] while in the western countries there is more focus on cannabis abuse due to its higher prevalence [4]. In terms of the importance of gateway drugs, Markwood has stated in his comprehensive theory of drug abuse prevention as a result of a holistic evaluation of all drug abuse risk factors, “in essence, the only way to achieve true primary prevention of abuse of postgateway drugs is to succeed at preventing or stopping use of gateway drug” [30]. According to growing popularity of waterpipe smoking among Iranian population more attention should be devoted to this epidemic by health policymakers.

Individual characteristics were also mentioned by the participants as one of drug abuse risk factors. In various etiological studies on addiction especially studies with an individualistic approach, there has been an emphasis on the role of personality, age of starting drug abuse [2, 6], and hereditary and genetic factors [26]. However, some believe that genetics is more involved with drug abuse rather than recreational drug abuse [31]. Culture-based research to give a clearer picture of personality traits of drug abusers is warranted.

Roles of the community and macroenvironment have also been noted in the interviews as risk factors of drug abuse. Poverty, neighbourhood, living in poor and polluted suburbs, and low level of leisure facilities have been identified as impacting factors.

In conflict theory of abuse, the role of social class, income, and locale have been revealed in drug abuse especially heavy drugs such as heroin and cocaine as it is mentioned in “just say poverty: what causes Crack and Heroin Abuse” [4]. However, some experts regard the neighbourhood effects as minimal [32].

Overall, considering the factors extracted from the stories of participants, we are able to report that identified risk factors are more in line with the principles mentioned in the social development theory [33] which states that youth would attach to drug-using peers if they did not have a strong enough bonding with their parents. This theory focuses on the concept of protective factors and risk factors, and it recognises a role for all levels of individual, family, peers, community, and the neighbourhood.

Amongst all the risk factors for drug abuse, this study has highlighted some based on the participants' story of their addiction. The impacts of family, peer pressure, and gateway drug abuse were identified as the most important of all factors.
